# Community-based integrated care for HIV, viral hepatitis and sexually transmitted infections, Thailand

**DOI:** 10.2471/BLT.25.293745

**Published:** 2025-11-21

**Authors:** Tanyaporn Wansom, Akaphot Thongmee, Salyavit Chittmittrapap, Tunyaluck Saraporn, Karuna Chavalertsakul, Nuntisa Chotirosniramit, Suta Pattarakijroongrueng, Thitisant Palakawong Na Ayuthaya, Viroj Verachai, Paisarn Traisirichok, Benjamas Intharabut, Nyan Linn, Rapeeporn Teuansiri, Kewalin Kulprayong, Arrini Waesateh, Sureena Lawseng, Tikumporn Chumwangwapee, Smith Pattarasuteewong, Nee Pudpong, Duangsamon Unchit, Jukraphan Photipap, Chutarat Wongsuwon, Nattapon Werapattanawong, Suhainong Smahoh, Pilanthana Sae-chee, Sakda Phueakchai, Prommin Kittikoonprasert, Pongsri Bootsan, Saranath Lawpoolsri, Philippe Creac’H, Stephen Mills, Pornsak Yoocharoen, Anchalee Avihingsanon, Nittaya Phanuphak, Arthorn Riewpaiboon, Nicolas Durier, on behalf of the C-Free Study Group.

**Affiliations:** a Dreamlopments Foundation, Phaholyothin Place, 9th Floor, Bangkok, 10400, Thailand.; b Center of Excellence in Hepatitis and Liver Cancer, Chulalongkorn University, Bangkok, Thailand.; c Chana District Hospital, Songkhla, Thailand.; d Sungai Kolok District Hospital, Narathiwat, Thailand.; e Research Institute for Health Sciences, Chiang Mai University, Chiang Mai, Thailand.; f Mae Ramat Hospital, Tak, Thailand.; g Public Health Center 28, Bangkok Metropolitan Administration, Bangkok, Thailand.; h New Step Clinic, Bangkok Metropolitan Administration, Bangkok, Thailand.; i Khon Kaen Hospital, Khon Kaen, Thailand.; j Raks Thai Foundation, Bangkok, Thailand.; k Association to Promote Access to Health and Social Support, Bangkok, Thailand.; l Care Team Songkhla, Songkhla, Thailand.; m Together, Narathiwat, Thailand.; n Thai Drug Users Network, Chiang Mai, Thailand.; o Give Hope, Mae Ramad, Tak, Thailand.; p Act Team Group, Khon Kaen, Thailand.; q Center of Excellence for Biomedical and Public Health Informatics, Mahidol University, Bangkok, Thailand.; r The Global Fund to Fight AIDS, Tuberculosis and Malaria, Geneva, Switzerland.; s FHI360, Bangkok, Thailand.; t Department of Disease Control, Ministry of Public Health, Nonthaburi, Thailand.; u HIV-NAT, Thai Red Cross AIDS and Infectious Diseases Research Centre, Bangkok, Thailand.; v Institute for HIV Research and Innovation, Bangkok, Thailand.; w Faculty of Pharmacy, Mahidol University, Bangkok, Thailand.

## Abstract

**Objective:**

To assess the impact of an integrated model of care in curing hepatitis C in people who use drugs in Thailand.

**Methods:**

The C-Free Study enrolled people with current or prior drug use and their partners in a prospective cohort at community drop-in centres providing harm reduction services. Participants were screened for human immunodeficiency virus (HIV), hepatitis C virus (HCV), hepatitis B virus (HBV) and sexually transmitted infections. Eligible participants with HCV infection received a 12-week course of sofosbuvir–velpatasvir. The main impact outcome was sustained virological response, measured 12 weeks after treatment completion.

**Results:**

Between June 2019 and April 2023, we enrolled 2871 participants in 10 sites across Thailand: 1601 (55.8%) had HCV antibodies; 1275 (44.4%) had active HCV infection; 846 (29.5%) had HIV; and 221 (7.7%) had HBV. Of 1134 participants with active HCV who started treatment with sofosbuvir–velpatasvir, 939 (82.8%) achieved a sustained virological response. Among 987 participants completing treatment, 95.1% achieved a sustained virological response. In multivariable analysis, age > 40 years (adjusted odds ratio, aOR: 1.63; 95% confidence interval, CI: 1.04–2.54) and poor treatment adherence (aOR: 0.06; 95% CI: 0.02–0.20) were associated with sustained virological response. Of 34 serious adverse events during treatment, six led to treatment discontinuation including five non-treatment-related deaths.

**Conclusion:**

Community-based HCV treatment of people who use drugs in Thailand, within harm reduction settings, is safe and effective. Integration of this strategy into national programmes could enhance HCV elimination in people who use drugs.

## Résumé

**Objectif:**

Évaluer l’impact d’un modèle intégré de soins pour le traitement de l’hépatite C chez les personnes qui consomment des drogues en Thaïlande.

**Méthodes:**

L’étude C-Free a recruté des personnes consommant ou ayant consommé des drogues et leurs partenaires dans une cohorte prospective dans des centres d’accueil communautaires proposant des services de réduction des risques. Les participants ont été soumis à un dépistage du virus de l’immunodéficience humaine (VIH), du virus de l’hépatite C (VHC), du virus de l’hépatite B (VHB) et des infections sexuellement transmissibles. Les participants éligibles infectés par le VHC ont reçu pendant 12 semaines l’association de sofosbuvir et de velpatasvir. Le principal résultat en termes d’impact était une réponse virologique soutenue, mesurée 12 semaines après la fin du traitement.

**Résultats:**

Entre juin 2019 et avril 2023, nous avons recruté 2 871 participants sur 10 sites dans toute la Thaïlande: 1 601 (55,8%) présentaient des anticorps contre le VHC; 1 275 (44,4%) avaient une infection active par le VHC; 846 (29,5%) étaient atteints du VIH; et 221 (7,7%) étaient atteints du VHB. Sur les 1 134 participants atteints d’une infection active par le VHC qui ont commencé un traitement par sofosbuvir et velpatasvir, 939 (82,8%) ont obtenu une réponse virologique soutenue. Parmi les 987 participants ayant terminé le traitement, 95,1% ont obtenu une réponse virologique soutenue. Au cours de l’analyse multivariée, un âge supérieur à 40 ans (odds ratio ajusté (aOR): 1,63; intervalle de confiance (IC) à 95%: 1,04–2,54) et une mauvaise observance du traitement (aOR: 0,06; IC à 95%: 0,02–0,20) étaient associés à une réponse virologique soutenue. Sur les 34 événements indésirables graves survenus pendant le traitement, six ont conduit à l’arrêt du traitement, dont cinq décès non liés au traitement.

**Conclusion:**

Le traitement sur base communautaire du VHC chez les personnes qui consomment des drogues en Thaïlande, dans le cadre de services de réduction des risques, est sûr et efficace. L’intégration de cette stratégie à des programmes nationaux pourrait améliorer l’élimination du VHC chez les personnes qui consomment des drogues.

## Resumen

**Objetivo:**

Evaluar el impacto de un modelo de atención integrada en la curación de la hepatitis C en personas que usan drogas en Tailandia.

**Métodos:**

El estudio C-Free inscribió a personas con consumo actual o previo de drogas y a sus parejas en una cohorte prospectiva en centros comunitarios de acogida que prestaban servicios de reducción de daños. Se realizó cribado de los participantes para detectar el virus de la inmunodeficiencia humana (VIH), el virus de la hepatitis C (VHC), el virus de la hepatitis B (VHB) y las infecciones de transmisión sexual. Los participantes elegibles con infección por VHC recibieron un tratamiento de 12 semanas con sofosbuvir-velpatasvir. El principal desenlace de impacto fue la respuesta virológica sostenida, medida 12 semanas después de finalizar el tratamiento.

**Resultados:**

Entre junio de 2019 y abril de 2023, se inscribieron 2871 participantes en 10 centros de Tailandia: 1601 (55,8%) tenían anticuerpos frente al VHC; 1275 (44,4%) presentaban infección activa por VHC; 846 (29,5%) tenían VIH; y 221 (7,7%) tenían VHB. De los 1134 participantes con infección activa por VHC que iniciaron tratamiento con sofosbuvir-velpatasvir, 939 (82,8%) lograron una respuesta virológica sostenida. Entre los 987 participantes que completaron el tratamiento, el 95,1% alcanzó una respuesta virológica sostenida. En el análisis multivariable, la edad > 40 años (razón de posibilidades ajustada, aOR: 1,63; intervalo de confianza del 95%, IC: 1,04-2,54) y la baja adherencia al tratamiento (aOR: 0,06; IC del 95%: 0,02-0,20) se asociaron con la respuesta virológica sostenida. De los 34 acontecimientos adversos graves ocurridos durante el tratamiento, seis llevaron a la interrupción del mismo, incluidos cinco fallecimientos no relacionados con el tratamiento.

**Conclusión:**

El tratamiento del VHC basado en la comunidad para personas que usan drogas en Tailandia, en el marco de servicios de reducción de daños, es seguro y eficaz. La integración de esta estrategia en los programas nacionales podría fortalecer la eliminación del VHC en personas que usan drogas.

## ملخص

**الغرض:**

تقييم أثر نموذج للرعاية المتكامل في علاج الالتهاب الكبدي سي لدى الأشخاص الذين يتعاطون ويحقنون المخدرات في تايلند.

**الطريقة:**

شملت دراسة "خالٍ من الالتهاب الكبدي سي" أشخاصًا يتعاطون المخدرات حاليًا أو سابقًا وشركائهم في مجموعة مستقبلية في مراكز الاستقبال المجتمعية التي تقدم خدمات الحد من الضرر. خضع المشاركون لفحص فيروس نقص المناعة البشرية (HIV)، وفيروس الالتهاب الكبدي سي (HCV)، وفيروس الالتهاب الكبدي ب (HBV)، والأمراض المنقولة جنسيًا. تلقى المشاركون المؤهلون المصابون بعدوى الالتهاب الكبدي سي دورة علاجية لمدة 12 أسبوعًا من العلاج باستخدام سوفوسبوفير-فيلباتاسفير. تمثلت نتيجة التأثير الأساسية في استمرار الاستجابة الفيروسية، والتي تم قياسها بعد 12 أسبوعًا من انتهاء العلاج.

**لنتائج:**

بين يونيو/حزيران 2019، وأبريل/نيسان 2023، قمنا بتسجيل 2871 مشاركًا في 10 مواقع في جميع أنحاء تايلند: 1601 (%55.8) كان لديهم أجسام مضادة للالتهاب الكبدي سي؛ وكان لدى 1275 (%44.4) من المشاركين عدوى نشطة بفيروس الالتهاب الكبدي سي؛ و846 (%29.5) مصابين بفيروس نقص المناعة البشرية؛ و221 (%7.7) مصابين بفيروس الالتهاب الكبدي ب. من بين 1134 مشاركًا مصابًا بفيروس الالتهاب الكبدي سي النشط، والذين بدأوا العلاج باستخدام سوفوسبوفير-فيلباتاسفير، حقق 939 (%82.8) منهم استجابة فيروسية مستدامة. ومن بين 987 مشاركًا أكملوا العلاج، حقق %95.1 منهم استجابة فيروسية مستدامة. في التحليل متعدد المتغيرات، ارتبط العمر > 40 عامًا (نسبة الأرجحية المعدلة، نسبة الأرجحية: 1.63؛ بفاصل ثقة مقداره %95: 1.04 إلى 2.54) وضعف الالتزام بالعلاج (نسبة الأرجحية المعدلة: 0.06؛ ‏بفاصل ثقة مقداره %95: 0.02 إلى 0.20) بالاستجابة الفيروسية المستدامة. من بين 34

**الاستنتاج:**

يُعدّ العلاج المجتمعي لفيروس الالتهاب الكبدي سي لدى الأشخاص الذين يتعاطون ويحقنون المخدرات في تايلند، ضمن إطار الحد من الضرر، آمنًا وفعالًا. ومن شأن تكامل هذه الاستراتيجية مع البرامج الوطنية أن يُعزز القضاء على فيروس الالتهاب الكبدي سي بين الأشخاص الذين يتعاطون ويحقنون المخدرات.

## 摘要

**目的:**

旨在评估治疗丙型肝炎时所采用综合照护模式对泰国毒品使用者或注射者的影响。

**方法:**

该项无化疗 (C-Free) 研究招募当前正在使用或之前使用过毒品的人员及其伴侣，以在提供减少危害服务的社区救助中心开展前瞻性队列研究。将参与者按人类免疫缺陷病毒 (HIV)、丙型肝炎病毒 (HCV)、乙型肝炎病毒 (HBV) 和性传播感染四类进行了筛选。符合条件的 HCV 感染者接受为期 12 周的索非布韦-维帕他韦治疗。主要影响指标是在治疗完成后第 12 周测得的持续病毒学应答情况。

**结果:**

在 2019 年 6 月至 2023 年 4 月期间，我们在泰国的 10 个站点共招募了 2,871 参与者：HCV 抗体携带者为 1,601 名（占 55.8%），活动性 HCV 感染者为 1,275 名（占 44.4%）；HIV 感染者为 846 名（占 29.5%）；以及 HBV 感染者为 221 名（占 7.7%）。在已开始接受索非布韦-维帕他韦治疗的 1,134 名活动性 HCV 感染者中，939 名（占 82.8%）实现了持续病毒学应答。在 987 名完成治疗的参与者中，95.1% 实现了持续病毒学应答。根据多变量分析结果，年龄超过 40 岁【调整后优势比 (aOR)：1.63；95% 置信区间 (CI)：1.04–2.54】和治疗依从性较差（aOR：0.06；95% CI：0.02–0.20）都会影响持续病毒学应答。在治疗期间出现的 34 例严重不良事件中，出现了 6 例治疗中止事件（包括五例非治疗相关死亡）。

**结论:**

针对泰国毒品使用者和注射者在减少危害服务中心采取基于社区的 HCV 治疗是一种安全有效的措施。

## Резюме

**Цель:**

Оценить влияние интегрированной модели медицинской помощи при лечении в Таиланде гепатита С у лиц, употребляющих и вводящих себе наркотики.

**Методы:**

В исследование C-Free были включены лица, употреблявшие наркотики на момент привлечения к участию или ранее, а также их партнеры. Исследуемая проспективная когорта была сформирована на базе общественных консультационных центров, предоставляющих услуги по снижению вреда. Участников обследовали на наличие вируса иммунодефицита человека (ВИЧ), вируса гепатита С (ВГС), вируса гепатита В (ВГВ) и инфекций, передаваемых половым путем (ИППП). Соответствовавшие критериям включения участники с инфекцией ВГС получали 12-недельный курс софосбувира-вельпатасвира. В качестве основного исхода воздействия служил устойчивый вирусологический ответ, измеренный спустя 12 недель после завершения лечения.

**Результаты:**

В период с июня 2019 г. по апрель 2023 г. в исследование был включен 2871 участник в 10 центрах на территории Таиланда: у 1601 (55,8%) были обнаружены антитела к ВГС, у 1275 (44,4%) наблюдалась активная инфекция ВГС, у 846 (29,5%) участников был найден ВИЧ, а у 221 (7,7%) – ВГВ. Из 1134 участников с активной инфекцией ВГС, которые начали получать софосбувир-вельпатасвир, 939 (82,8%) достигли устойчивого вирусологического ответа. Среди 987 участников, завершивших лечение, 95,1% достигли устойчивого вирусологического ответа. В ходе мультивариантного анализа было обнаружено, что возраст старше 40 лет (скорректированное отношение шансов, сОШ: 1,63; 95%-й доверительный интервал, ДИ: 1,04–2,54) и плохая приверженность лечению (сОШ: 0,06; 95%-й ДИ: 0,02–0,20) ассоциировались с устойчивым вирусологическим ответом. Из 34 серьезных нежелательных явлений, имевших место в ходе лечения, шесть привели к прекращению терапии, в том числе в пяти случаях причиной стала смерть, не связанная с лечением.

**Вывод:**

На базе общественных организаций, предоставляющих услуги по снижению вреда в Таиланде, лечение ВГС у лиц, которые употребляют и вводят себе наркотики, является безопасным и эффективным. Интеграция этих стратегий в национальные программы может способствовать ликвидации ВГС у лиц, употребляющих и вводящих себе наркотики.

## Introduction

People who use drugs are particularly affected by hepatitis C virus (HCV), and account for more than half of global human immunodeficiency virus (HIV) and HCV coinfections.^1^ While direct antiviral agents can cure HCV in over 95% of cases, treatment of people who inject drugs is limited.^2^^–^^4^ In Thailand, 700 000 people are estimated to be living with HCV; in people who inject drugs, HCV prevalence ranges from 20% to 80%.^5^^–^^8^

Although sofosbuvir–velpatasvir became the preferred HCV treatment for Thailand’s national HCV programme in 2021, there are burdensome eligibility criteria including: (i) HCV ribonucleic acid (RNA) should be > 5000 IU/mL; (ii) liver fibrosis should be moderate or advanced; and (iii) prescription must be made by specialist physicians. Active substance use was an exclusion criterion. For people who use drugs, many report that stigma and discrimination by health workers persists, further limiting access to treatment opportunities.^9^

To overcome these barriers, we embedded the C-Free study intervention (hereafter called C-Free), offering testing and treatment of viral hepatitis and HIV, in drop-in centres providing harm reduction services for people who use drugs. We aimed to assess the success of this integrated model of care in curing HCV in people who use drugs, and their partners, across Thailand.

## Methods

### Study sites

We conducted a prospective cohort study among people attending drop-in centres in Thailand between May 2019 and April 2023. Before C-Free, these centres offered no clinical services, only syringe exchange services and peer and social support programmes.

We embedded C-Free in 10 drop-in centres, three located in Bangkok and seven in other provinces across Thailand. Eight drop-in centres were run by partner community-based organizations and two were community health clinics run by the Bangkok Metropolitan Administration. The study sponsor procured all necessary equipment and tests, and C-Free nurses carried out all study procedures in a designated room at each drop-in centre. 

### Participants

Eligible participants were 18 years and older and self-reported either current or past drug use, or being the sexual or life partner of a person who currently or previously reported such use. Outreach workers from the drop-in centre recruited participants for the C-Free study. Some participants were recruited by word-of-mouth or referred by health facilities. 

### Procedures

All participants enrolled in the study were offered HIV, hepatitis B virus (HBV) and HCV blood tests. External quality assurance for each blood test was done annually. HIV testing followed the Thai national guidelines.^10^ Study physicians from partner hospitals held weekly clinics at the drop-in centres to assess eligibility for HCV treatment, and prescribe and monitor treatment in the HCV study.

Participants with reactive HIV, HCV and/or HBV (hepatitis B surface antigen, HBsAg) serology had molecular testing done at the drop-in centre using GeneXpert® (Cepheid, Sunnyvale, United States of America). Participants with HIV and/or HBV infection were referred to the national health system. Participants who tested negative for both HBsAg and hepatitis B surface antibody (HBsAb) were offered the HBV vaccine (rDNA, Serum Institute of India, Pune, India) at 0, 1 and 6 months. Participants with negative HIV or HCV antibody tests, or negative HCV RNA after a positive HCV antibody test, were offered retesting every 6 months.

Participants with active HCV, defined as HCV RNA greater than the lower limit of detection (10 IU/mL), were potentially eligible for enrolment in the HCV treatment arm. Exclusion criteria for HCV treatment were: (i) history of prior treatment failure with a sofosbuvir-containing regimen (self-reported or in medical records); (ii) decompensated cirrhosis (determined clinically and through aspartate aminotransferase to platelet ratio (APRI) > 2 and Child-Pugh B and C scores);^11^ (iii) hepatocellular carcinoma (from medical records or ultrasound for participants with APRI > 2); (iv) estimated glomerular filtration rate < 30 mL/min; and (v) pregnancy (pregnancy test at screening). Participants eligible for HCV treatment were offered a generic fixed-dose combination of sofosbuvir–velpatasvir (MyHep All®, Viatris, India) once daily for 12 weeks. 

Participants receiving HCV treatment had scheduled visits with study physicians at weeks 0, 4, 8, 12 and 24. C-Free nurses dispensed sofosbuvir–velpatasvir at weeks 0, 4 and 8 with pill counts done at each subsequent visit to assess treatment adherence. Safety laboratory tests, initially required at week 4, were made optional in September 2019. For participants reinfected with HCV, defined as detectable HCV RNA ≥ 6 months after a sustained virological response, sofosbuvir–velpatasvir retreatment was offered.

We added testing for sexually transmitted infections to the protocol in 2021. All participants were screened with Determine Syphilis TP (Abbott, Minato, Japan). Reactive participants had a rapid plasma reagin test and participants with a titre of 1:8 or more were referred for syphilis treatment. Testing for *Neisseria gonorrhoea* and *Chlamydia trachomatis* was offered to a subset of participants who had either a positive syphilis test or reported condomless intercourse with multiple partners in the past year. Self-collected swabs were pooled by the individual and tested using GeneXpert® for *N. gonorrhoea* and *C. trachomatis*. Participants diagnosed with either or both infections were treated with ceftriaxone and azithromycin at the centre.

### Outcome measures

Primary outcomes included prevalence of HIV, HBV and HCV, and for participants with HIV, the percentage taking antiretroviral treatment (ART) and reaching virological suppression, HIV RNA < 40 copies/mL measured at least once annually.

For the HCV treatment study, the primary impact outcome was sustained virological response at 12 weeks after treatment completion, defined as HCV RNA less than the lower limit of quantification by GeneXpert® testing (< 10 IU/mL). The primary safety outcome was occurrence of serious adverse events. In the participants achieving sustained virological response, HCV RNA was measured every 6 months to monitor for reinfection.

### Statistical analysis

We calculated the prevalence of HIV, HBV and HCV and 95% confidence intervals (CI) based on test results at enrolment. We assessed HIV and HCV incidence as rates per 100 person-years of follow-up in participants with negative test results and seroconversion during follow-up. We estimated the proportion of participants achieving sustained virological response (with 95% CI) in the overall population and by age, drug use status, treatment adherence and HIV coinfection. We calculated sustained virological response both in an intent-to-treat analysis for all participants prescribed HCV treatment, and in a per-protocol analysis for participants completing treatment and attending the sustained virological response visit. We calculated crude odds ratios (OR) to assess factors associated with sustained virological response. We included factors with *P* < 0.05 in a multivariable model to determine adjusted ORs (aOR) after removing closely associated predictors to avoid collinearity. We used SAS 9.4 (SAS Institute, Cary, USA) for all analyses.

### Ethical considerations

The study was registered with the Thai Clinical Trials Registry (TCTR20171115002) and approved by the following ethics committees and regulatory body: Thai Central Research Ethics Committee; Chulalongkorn University; Chiang Mai University; Bangkok Metropolitan Administration; FHI360; and the Thai Food and Drug Administration. All participants provided written informed consent before enrolment. For participants unable to read and/or write, we gave a verbal explanation of the study, and an appropriate witness confirmed its accuracy before participants gave their consent with their fingerprint with the witness signing.

## Results

### Full cohort

#### Characteristics

In all, 2871 participants from 50 provinces were enrolled in C-Free, with 37.2% (1068) from metropolitan Bangkok. Median age was 41 years (interquartile range, IQR: 32–47); 84.8% (2434) were male; and 19.6% (478/2434) of men reported having sex with other men (Table 1). Most participants (79.6%; 2285) were recruited by community outreach workers and 95.5% (2742) were Thai. With regard to drug use, 62.1% (1783) of participants reported current drug use (defined as drug use at least once in the past year) by any route of administration, with 34.3% (984) currently injecting drugs.

**Table 1:** Characteristics of the participants at enrolment visit, by study group, Thailand, 2019–2023

Characteristic	Complete cohort (*n* = 2871)	Treatment-eligible (*n* = 1134)
**Age, years**		
Median (IQR)	41 (32–47)	43 (35–48)
Range	18–77	18–74
**Self-reported gender, no (%)**		
Male	2434 (84.8)	1030 (90.8)
Female	410 (14.3)	95 (8.4)
Transgender	27 (0.9)	9 (0.8)
**Participant recruitment, no. (%)**		
Community outreach worker	2285 (79.6)	787 (69.4)
C-Free participant	196 (6.8)	70 (6.2)
Hospital or physician	164 (5.7)	120 (10.6)
No answer or self-referred	226 (7.9)	157 (13.8)
**Current drug use (any route of administration), no. (%)**	1783 (62.1)	607 (53.5)
**Injected drug in the past year, no. (%)**	984 (34.3)	471 (41.5)
**History of prior injecting drug use, no. (%)**	872 (30.4)	511 (45.1)
**Current methadone use, no. (%)**	789 (27.5)	379 (33.4)
**Alcohol use, no. (%)**		
Occasional use	1165 (40.6)	441 (38.9)
Regular use	459 (16.0)	144 (12.7)
Prior alcohol use	1245 (43.4)	537 (47.4)
Never	461 (16.1)	156 (13.8)
**HIV antibody reactive, no. (%)**	846 (29.5)	521 (45.9)
Taking antiretroviral therapy	785 (92.8)	511 (98.1)
HIV RNA < 40 copies/mL (% among people tested)	584/793 (73.6)	456/503 (90.7)
**HBV, no. (%)**		
HBsAg reactive	221 (7.7)	59 (5.2)
HBsAb reactive	900 (31.3)	404 (35.6)
HBsAg and HBsAb non-reactive	1750 (61.0)	671 (59.2)
Started HBV vaccination	1513/1750 (86.5)	662/671 (98.7)
Completed HBV vaccination	890/1513 (58.8)	527/662 (79.6)
**HCV **		
HCV antibody reactive, no. (%)	1601 (55.8)	1134 (100)
HCV RNA > lower limit of detection % of HCV antibody reactive, no. (%)	1275/1601 (79.6)	1134 (100)
HCV RNA log10 IU/mL, median (IQR)	NA	6.42 (4.72–6.84)
**Coinfections, no. (%)**		
HIV and HBV	23/846 (2.7)	NA
HIV and HCV	587/846 (69.4)	556 (49.0)
HBV and HCV	NA	50 (4.4)
HIV, HBV and HCV	33/846 (3.9)	NA
**APRI score, no. (%)**		
0–1.5	NA	943 (83.2)
> 1.5– < 2.0	NA	56 (4.9)
≥ 2.0	NA	135 (11.9)
**Syphilis, no. (%) **		
Tested	1761 (61.3)	NA
Rapid plasma reagin reactive (titre 1:8 or higher)	99/1761 (5.6)	NA
***Chlamydia trachomatis* and *Neisseria gonorrhoea*, no. (%) **		
Tested	353 (12.3)	NA
Positive for both infections	27/353 (7.6)	NA
Positive *C. trachomatis* only	66/353 (18.7)	NA
Positive *N. gonorrhoea* only	46/353 (13.0)	NA

APRI: aspartate aminotransferase to platelet ratio; HBV: hepatitis B virus; HBsAb: hepatitis B surface antibody; HBsAg: hepatitis B surface antigen; HCV: hepatitis C virus; HIV: human immune deficiency virus; IQR: interquartile range; NA: not applicable; RNA: ribonucleic acid.

#### HIV, HBV and HCV

Of the 2871 participants, 846 (29.5%) were living with HIV; 92.8% (785/846) knew they were HIV positive before entering the study. Of 2086 participants tested, 61 (2.9%) were newly diagnosed with HIV. Among the participants with HIV, 92.8% (785/846) reported taking ART. Of 793 participants with HIV RNA results, 584 (73.6%) had HIV RNA < 40 copies/mL at some time during the study. Of 209 participants with detectable HIV RNA, 52 (24.9%) had their viral load subsequently decreased to undetectable levels. Overall, 73.3% (620/846) of participants with HIV had active HCV, compared with 32.3% (655/2025) without HIV.

7.7% (221) of the participants tested positive for HBsAg; 53.5% (61/114) had HBV deoxyribonucleic acid > 40 IU/mL. In all, 31.3% (900) tested positive for HBsAb, indicating immunity to HBV. Of 1750 (61.0%) participants negative for HBsAg and HBsAb, 1513 (86.5%) started HBV vaccination, with 890 (58.8%) completing the course.

Regarding HCV, 55.8% (1601) of the participants had HCV infection. Of the participants with positive HCV antibody, 79.6% (1275/1601) had active HCV infection (detectable HCV RNA), equivalent to 44.4% of all cohort participants.

#### Sexually transmitted infections

Of the full cohort, 61.3% (1761) were screened for syphilis: 173 (9.8%) had reactive testing and 150 (86.7%) were men who have sex with men. A total of 99 participants (57.2% of participants with reactive treponemal test) had a rapid plasma reagin titre of 1:8 or higher. Of 353 (12.3%) participants tested for *C. trachomatis* and/or *N. gonorrhoea*, 66 (18.7%) were positive for *C. trachomatis*, 46 (13.0%) for *N. gonorrhoea* and 27 (7.6%) for both. Most participants (286) tested for sexually transmitted infections were men who have sex with men.

### HCV treatment cohort

#### Characteristics

**Fig. 1:**
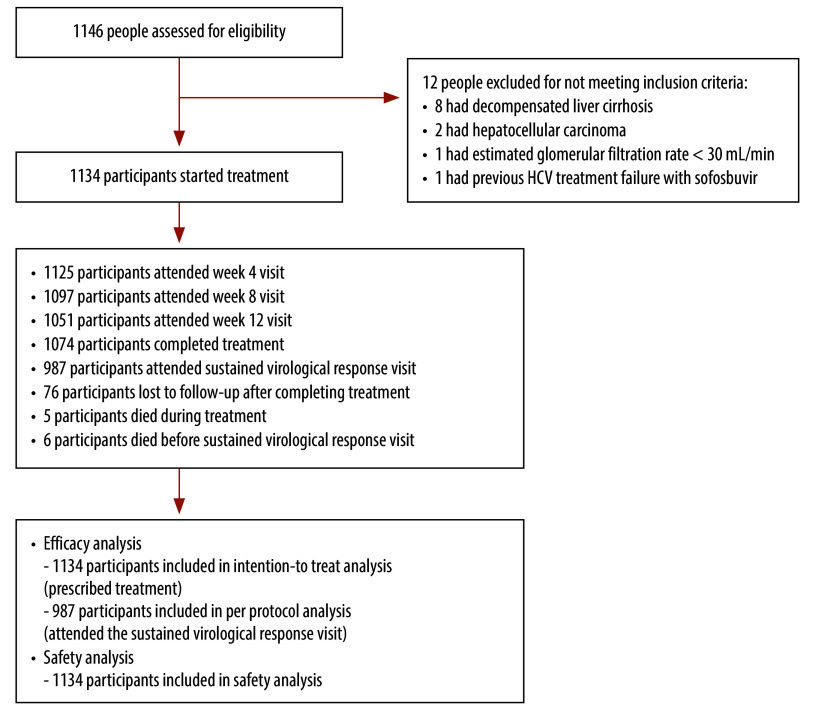
Flowchart of participants treated for hepatitis C virus infection, Thailand, 2019–2023

Of 1275 participants with active HCV, 1134 (88.9%) met all eligibility criteria and received HCV treatment (Fig. 1). Median HCV RNA was 6.42 log 10 IU/mL (IQR: 4.72–6.84) and 135 (11.9%) had an APRI ≥ 2.0. Only 1.5% (17) of the HCV participants had treatment adherence ≤ 90%.

#### Outcomes

Of the 1134 participants on treatment, 939 (82.8%; 95% CI: 80.0–85.0) achieved a sustained virological response in the intent-to-treat analysis. When excluding the 136 participants who did not attend the sustained virological response visit, or the 11 who died before sustained virological response, the per protocol sustained virological response among 987 participants attending the response visit was 95.1% (95% CI: 93.6–96.4). Treatment failure was documented in 4.9% (48/987) of participants. For the 26 participants who had consented for their blood specimens to be stored, HCV genotyping was conducted at baseline and the sustained virological response visit. There were 16 concordant genotypes at baseline and at sustained virological response, suggesting treatment failure rather than early reinfection. The genotype breakdown was: six samples with genotype 3a, five with 6/6n, two with 3/3b, two with 1a and one with 1b. We referred participants with treatment failure to public hospitals for further management.

#### Factors associated with sustained virological response

**Fig. 2:**
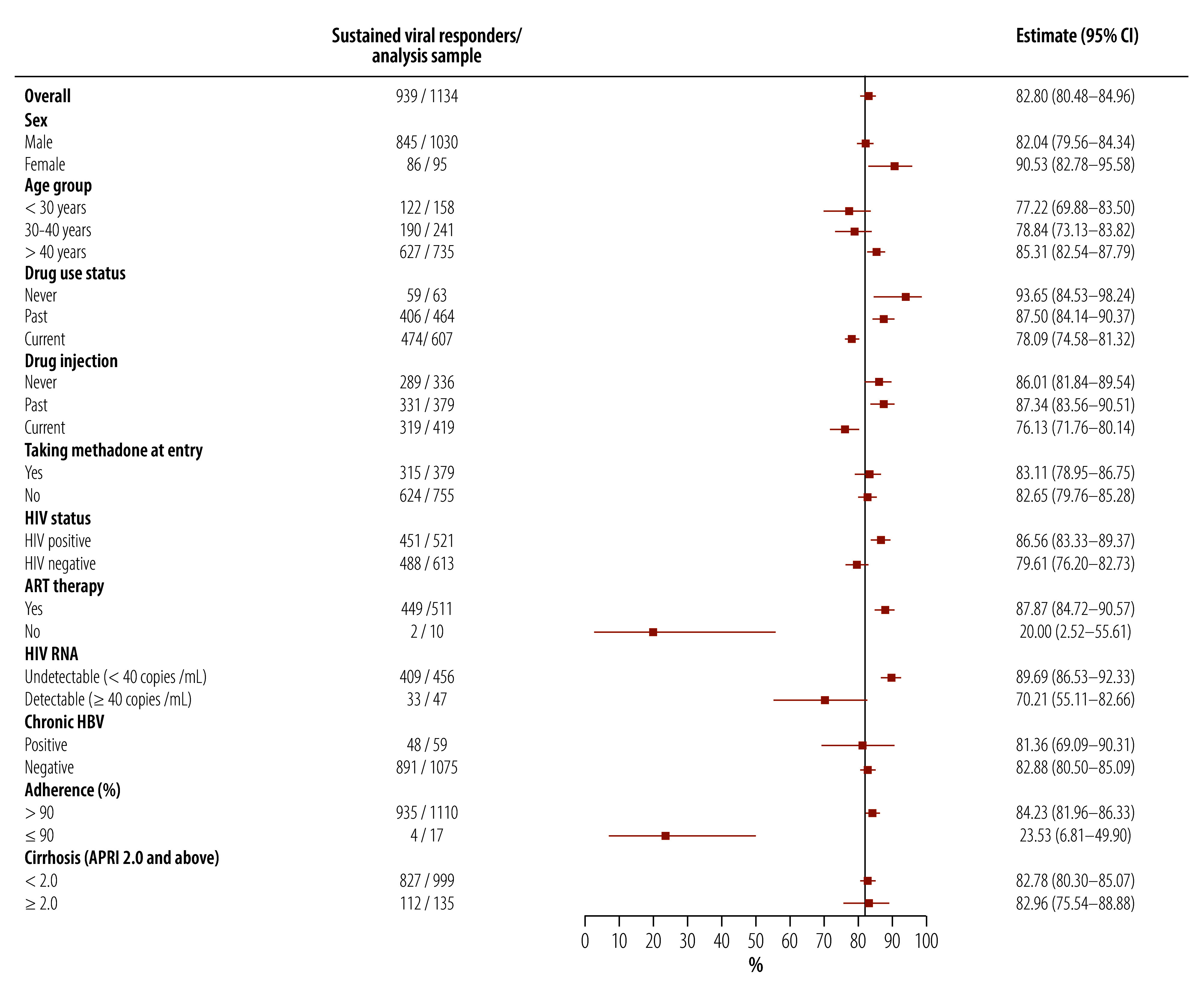
Sustained virological response following hepatitis C virus treatment, by participant characteristic, Thailand, 2019–2023

APRI: aspartate aminotransferase to platelet ratio; ART: antiretroviral therapy; HBV: hepatitis B virus; HIV: human immune deficiency virus; RNA: ribonucleic acid.

Of 135 participants with compensated liver cirrhosis, 112 (83.0%) achieved sustained virological response as per intent-to-treat analysis, as did 82.8% (827/999) without liver cirrhosis. Sustained virological response was achieved in 86.6% (451/521) and 79.6% (489/613) of participants with and without HIV, respectively (Fig. 2). In univariate analysis (Table 2), sustained virological response was significantly associated with former or no drug use compared with current drug use, OR: 1.96 (95% CI: 1.40–2.75) for former drug users and OR: 4.13 (95% CI: 1.48–11.58) for never users. Participants older than 40 years, having HIV, taking ART and having undetectable HIV RNA were also associated with sustained virological response; poor treatment adherence was negatively associated with sustained virological response. In multivariable analysis, only participants older than 40 years (aOR: 1.63; 95% CI: 1.04–2.54) and poor treatment adherence (aOR: 0.06; 95% CI: 0.02–0.20) were significantly associated with sustained virological response.

**Table 2:** Variables associated with sustained virological response, Thailand, 2019–2023

Variable	OR (95% CI)	
	Crude	Adjusted
**Sex^a^**		
Male	Ref.	Ref.
Female	2.09 (1.03–4.23)	1.50 (0.67–3.38)
**Age group, years**		
< 30	Ref.	Ref.
30–40	1.10 (0.68–1.78)	1.10 (0.67–1.81)
> 40	1.71 (1.12–2.62)	1.63 (1.04–2.54)
**Drug use**		
Never	4.13 (1.48–11.59)	2.21 (0.70–6.99)
Past	1.96 (1.40–2.75)	1.37 (0.63–3.00)
Current	Ref.	Ref.
**Drug injection**		
Never	1.93 (1.32–2.82)	1.35 (0.85–2.14)
Past	2.16 (1.48–3.15)	1.36 (0.56–3.29)
Current	Ref.	Ref.
**Methadone use at entry**		
Yes	Ref.	NA
No	0.97 (0.70–1.34)	NA
**HIV infection**		
Yes	1.65 (1.20–2.27)	1.39 (0.99–1.95)
No	Ref.	Ref.
**On antiretroviral treatment**		
Yes	Ref.	NA^c^
No	0.04 (0.01–0.17)	NA
**Detectable HIV RNA (≥ 40 copies/mL) **		
No	3.69 (1.84–7.39)	NA^c^
Yes	Ref.	NA
**HBV infection**		
Yes	Ref.	NA
No	1.11 (0.57–2.18)	NA
**Treatment adherence**		
> 90%	Ref.	Ref.
≤ 90%	0.06 (0.02–0.18)	0.06 (0.02–0.20)
**Cirrhosis^b^**		
No	0.99 (0.61–1.59)	NA
Yes	Ref.	NA

CI: confidence interval; HBV: hepatitis B virus; HIV: human immune deficiency virus; RNA: ribonucleic acid; OR: odds ratio; Ref.: reference category.

^a^ Transgender people were not included in this analysis as their number was small.

^b^ Based on aspartate aminotransferase to platelet ratio ≥ 2.0.

^c^ This variable was not included in the multivariable analysis as it is highly correlated with HIV infection.

Note: We removed participants with missing values in any individual parameter from the analysis.^.^

#### Primary safety outcomes

Of the 1134 participants starting HCV treatment, 209 (18.4%) experienced a grade 3 or 4 adverse event during follow-up (Table 3). Additionally, 3.0% (34/1134) of these participants experienced serious adverse events, including five deaths, none of which were related to HCV treatment. Three deaths were related to drug use, one was due to complications of end-stage liver disease, and one to sepsis and acute renal failure. Two adverse events (rash and chest discomfort) and one serious adverse event (deep venous thrombosis) resulted in treatment discontinuation.

**Table 3:** Adverse events in participants treated for HCV infection, Thailand, 2019–2023

Event	No. (%), *n* = 1134
**At least one grade 3 or 4 adverse event or serious adverse event**	209 (18.4)
**Serious adverse event while on HCV study medication**	34 (3.0)^a^
**Adverse event or serious adverse event leading to discontinuation of HCV study medication**	8 (0.7)^a^
**Death**	42 (3.7)
During treatment	5 (0.4)
After completing treatment but before sustained virological response	6 (0.5)
Lost to follow-up before death	5 (0.4)
After sustained virological response	26 (2.3)

HCV: hepatitis C virus.

^a^ Including five deaths.

Of participants prescribed HCV treatment, 3.7% (42/1134) died: five deaths occurred while participants were receiving HCV treatment, six occurred before the sustained virological response visit and 26 occurred after participants achieved sustained virological response. The other five deaths occurred in participants who discontinued treatment and were lost to follow-up before death. The commonest causes of death were infection in 12 participants, trauma or accidents in nine and drug-related deaths in six participants. Two deaths were related to gastrointestinal haemorrhage and one to complications of cirrhosis.

#### Reinfections

Among participants who achieved sustained virological response and were retested at least once for HCV RNA (513 participants), 35 were reinfected, giving a reinfection incidence rate of 3.75 per 100 person-years. Of these participants, three spontaneously cleared and 15 chose to be re-treated with sofosbuvir–velpatasvir in C-Free. Of the remaining 17 participants, six were subsequently retreated in the current C-Free2 study, one developed hepatocellular carcinoma and was treated in hospital and 10 followed up elsewhere or were lost to follow-up. Of the 15 re-treated participants, 14 achieved a sustained virological response.

### HIV and HCV incidence

Among participants initially negative for HIV, seven became HIV reactive on retesting, an HIV incidence of 0.59 per 100 person-years. Of participants initially negative for HCV antibody or positive for HCV antibody but negative HCV RNA, 44 had detectable HCV antibody or HCV RNA on retesting, an HCV incidence of 1.26 per 100 person-years.

## Discussion

As an innovative, community-based model of care, C-Free linked clinical services for high-burden diseases with strong harm reduction services implemented by community-based organizations. This initiative created people-centred, integrated and decentralized services in line with World Health Organization (WHO) recommendations on simplified service delivery for HCV care.^12^ C-Free met a considerable community need and provided effective measures for people who inject or use drugs and their partners. In the intent-to-treat analysis, about four fifths had a sustained virological response and in the per protocol analysis this proportion was 95.1%. These results are similar to a Vietnamese cohort with a sustained virological response of 97.2% (629/647), and an Italian cohort with a sustained virological response of 89.2% (66/74) among people who recently injected drugs and an overall sustained virological response of 94.4% (338/358).^13^^,^^14^ These cohorts treated patients at either a hospital (Viet Nam) or specialized outpatient clinic (Italy). C-Free achieved similar sustained virological response rates using a one-stop model for HCV diagnosis and treatment at drop-in centres.

In addition to curing HCV, C-Free identified participants with uncontrolled HIV infection and re-engaged them in care. Furthermore, 221 participants were diagnosed with chronic HBV infection and referred to the national programme, while 1750 had no immunity to HBV, most of whom chose to be vaccinated against HBV. Sexually transmitted infections were common: 99 had active syphilis infection and 139 participants with high-risk sexual behaviour tested positive for *C. trachomatis* and/or *N. gonorrhoea*.

The community-based organizations partnered with C-Free provide most of the syringe services in Thailand and recruited most of the C-Free participants through direct outreach. Community workers provided essential support to C-Free participants to encourage adherence to HCV treatment, follow-up appointments, home delivery of medications (especially during the coronavirus disease 2019 (COVID-19) pandemic) and links to health care. While the study included both past and current drug users, the reinfection rate of 3.75 per 100 person-years was lower than the rate in a community study of people who inject drugs in Viet Nam, with a reinfection rate of 4 per 100 person-years.^13^ Our rate is also lower than a recent meta-analysis that reported a reinfection rate of 5.9 per 100 person-years (95% CI: 4.1–8.5) in people reporting recent drug use.^15^

Although HCV treatment is covered under Thai government insurance, access is limited due to low rates of disease awareness, exclusion of people who use drugs from treatment until 2023, and the use of pegylated interferon to treat HCV genotype 3 until 2021. C-Free interim results in 2020 and 2021 contributed to positive developments in the national HCV programme. In February 2021, sofosbuvir–velpatasvir was designated the preferred treatment for all people with HCV. In August 2022, the National Essential Drug List Subcommittee recommended revisions to conditions of treatment with sofosbuvir–velpatasvir, including supporting treatment by trained general practitioners in all district hospitals and removal of substance use as an ineligibility criterion.

Of the C-Free participants in our study, one fifth were men who have sex with men. As awareness of HCV treatment at C-Free has grown, other community-based organizations serving lesbian, gay, bisexual, transgender, intersex and queer individuals and sex workers began screening clients for HCV and referring participants to C-Free. Men who have sex with men had the highest rates of reactive syphilis antibody compared with the overall rate, and of these men, 44.4% (127/286 tested) had chlamydia and/or gonorrhoea. These results underscore the importance of recognizing the interconnection of risk when addressing syndemics related to high-risk sexual behaviours combined with drug use.^16^^,^^17^

About a quarter of the C-Free cohort had both HIV and HCV. Most participants living with HIV were aware of their diagnosis, but C-Free supported them by identifying individuals with unsuppressed infection, providing adherence support and assisting with re-engagement in care. Indeed, about a quarter of participants with detectable HIV viral load while in C-Free subsequently achieved suppression of the virus. Participants whose HIV infection was not suppressed were eligible for HCV treatment in C-Free, but they were counselled on the lower likelihood of a sustained virological response to their HCV infection. In multivariable analysis, the only factors significantly associated with a sustained virological response were participants older than  40 years and a treatment adherence of < 90%. These factors are consistent with other studies that have shown good adherence strongly influences sustained virological response in people who inject drugs.^18^^,^^19^ Strategies to support adherence for young people and people currently using drugs are urgently needed.

As the mean age of our cohort was 40 years, most participants had not been vaccinated against HBV, as universal HBV vaccine coverage in infants started in Thailand in 1992^20^ and vaccination of adults is not covered by the national health insurance programme. WHO now recommends vaccination of adults at higher risk of HBV infection, including people who use drugs, men who have sex with men, and people with HCV and HIV.^21^ Most non-immune participants in our study started vaccination, with more than half completing the course.

Our study has some limitations. C-Free was a single-country study, so our findings may not be generalizable to other country settings. During the COVID-19 pandemic, we had to adapt procedures by allowing postal or home delivery of medication and assessment or pill counts through telemedicine. Because Thailand’s national HIV programme primarily used efavirenz-based regimens during the study, participants with HIV had to be referred to their HIV health worker to switch to an antiretroviral regimen compatible with sofosbuvir–velpatasvir. Some participants died or were lost-to-follow-up before HCV treatment could be started. During the study, direct outreach to HIV health workers and provincial public health authorities helped increase awareness of C-Free and the need to change antiretroviral medication and boost referrals.

A strength of C-Free was its size; the large longitudinal cohort study included participants from 50 provinces throughout Thailand. The study has provided a wide range of epidemiological information on multiple infections. C-Free also provides value for money in diagnosing and treating people who use drugs. We found that the cost per HCV diagnosis and per HCV cure in our community-based clinical care model was, respectively, 3866 Thai baht (฿; 122 United States dollars, US$) and ฿28 821 (US$ 907).

To conclude, the C-Free study provides important evidence about the impact of community-based integrated harm-reduction and clinical services for populations most at risk of HCV infection and other infectious diseases, who are also least likely to be diagnosed and treated in traditional health-care settings. With high cure rates and low reinfection, integrated harm reduction and clinical services for people who use drugs can contribute to elimination of hepatitis C. Additionally, these integrated services allowed for case-finding and treatment of sexually transmitted infections and hepatitis B, and supported re-engagement in HIV care. Given these multiple benefits, we strongly advocate for reimbursement of community-based testing and expansion of government-supported treatment to make national programmes for HIV, hepatitis and sexually transmitted infections sustainable as recommended by WHO.^22^
